# Cross-Sectional Evaluation of Food Items Preferred by Adolescents under the Influence of Television Advertisements

**DOI:** 10.34172/jrhs.2022.74

**Published:** 2021-12-28

**Authors:** Derya Dikmen, Ezgi Bellikci-Koyu, Kubra Isgin-Atici, Elif Inan-Eroglu, Asli Akyol, Aylin Ayaz, Reyhan Nergiz-Unal, Zehra Buyuktuncer

**Affiliations:** ^1^Department of Nutrition and Dietetics, Faculty of Health Sciences, Hacettepe University, Ankara, Turkey; ^2^Izmir Katip Celebi University, Faculty of Health Sciences, Department of Nutrition and Dietetics, Izmir, Turkey; ^3^Charles Perkins Centre, School of Health Sciences, Faculty of Medicine and Health, University of Sydney, NSW, Australia

**Keywords:** Advertisements, Adolescent, Food, Television, World Health Organization

## Abstract

**Background:** Food and beverage advertisements on television play a significant role in food preferences, especially among children and adolescents. This study aimed to evaluate foods and beverages advertised on television and purchased by adolescents or their families using the World Health Organization (WHO) nutrient profiling model.

**Study design:** A cross-sectional study.

**Methods:** This cross-sectional study was performed on 2,699 students (1380 males and 1319 females) aged 11-16 in Ankara, Turkey, in 2015. Socio-demographic characteristics, television-viewing habits, and the tendency to purchase foods and beverages under the influence of TV advertisements were recorded. The body weight and height were measured by the researchers. All reported food and beverage items (n = 284) were evaluated and classified as permitted or not permitted to advertise, according to the WHO nutrient profile model (2015).

**Results:** The majority (69.8%) of students were underweight/normal weight, whereas 13.3% and 16.9% were classified as overweight and obese, respectively. A total of 69.6% of adolescents declared that they were influenced by food advertisements, and 66.4% bought those foods. The most purchased products included cakes and sweet biscuits (63.8%), chocolate and confectionery (44.9%), savory snacks (39.6%), and soft drinks (25.4%). Only 8.5% of all the advertised products (n = 284) were permitted to be advertised, according to the WHO nutrient profile model (2015). Dairy products, meat products, grains, fruits and vegetables, soup, and some traditional Turkish foods (e.g., cig kofte and Turkish pizza) were permitted. The permitted products were preferred by only 13.6% of the adolescents.

**Conclusions:** Unhealthy foods are advertised on television for adolescents, and food advertisement management may be an essential strategy to provide healthy food choices.

## Background

 Adolescence is a particular period of life in terms of high growth rate and developing lifestyle behaviors that would continue throughout one’s lifetime. Obesity is one of the common health problems for adolescents and has been increasing internationally. Childhood and adolescence obesity is associated with such chronic diseases as cardiovascular diseases, type 2 diabetes, and some types of cancers and dental caries and has been accepted as the primary global public health concern, accordingly.^1^ Based on the estimation of the World Health Organization (WHO), over 340 million children and adolescents aged 5-19 years were overweight or obese in 2016.^2^ According to the Turkey Nutrition and Health Survey, the prevalence of obesity and overweight in children aged 6-18 and adolescents were 8.2% and 14.3%, respectively.^3^

 Obesity etiology is highly complex and includes genetic, environmental, physiological, psychological, and socioeconomic factors. Due to the modifiable nature of environmental factors of obesity, including diet, physical activity, and exposure to an obesogenic environment (through food advertisements via multimedia), these factors play an essential role in obesity prevention and management.^4,5^The marketing of nutrient-poor foods has raised concern over the increased prevalence of obesity among children and adolescents.^6,7^ Food marketing has a direct impact on nutritional knowledge, food preference, purchasing behaviors, consumption patterns, and diet-related health of people exposed to this advertisements.^8^ Despite the growth of online marketing systems, television (TV) is still the primary media tool involved in food and beverages promotion.^9,10^

 TV food advertising is a type of food marketing that targets children and adolescents in many countries. Food advertising encourages the consumption of low nutrient-dense food and beverages high in sugar, fat, and salt.^11-14^ TV marketing involves persistent techniques that promote regular consumption and recurrent purchase of these foods.^15,16^ Constant exposure to TV advertisements of unhealthy foods can lead to unhealthy eating patterns.^17^ Population-wide interventions are required to reduce dietary risk factors of non-communicable diseases; however, limited exposure to unhealthy food marketing in children and adolescents has been introduced as a cost-effective strategy by WHO.^18^

 Nutrient profiling is a tool for categorizing foods according to their nutritional composition.^20^ Different nutrient profiling models have been developed by academics, governmental and non-governmental organizations, and the food industry. Nutrient profiling models aim to regulate food marketing, identify foods eligible for health, and guide consumers to healthy food choices.^21^ WHO confirmed nutrient profiling as a valid method with interventions designed to increase the overall nutritional quality of diets.^22^ WHO Regional Office for Europe has developed the European nutrient profile model to improve the nutritional quality of foods marketed to children.^23^ Although nutrient profiling has been used to regulate food marketing to children in many countries,^24,25^ it is an emerging issue in Turkey. Recent regulation has been introduced in Turkey to restrict TV advertisements during children’s programs in 2018. This model is an adaptation of the WHO European nutrient profiling model to provide a common perspective in nutrient profiling of foods in the region. This study aimed to assess the eligibility of foods and beverages advertised on TV and purchased by adolescents using the WHO European nutrient profile model.

## Methods

**Figure 1:**
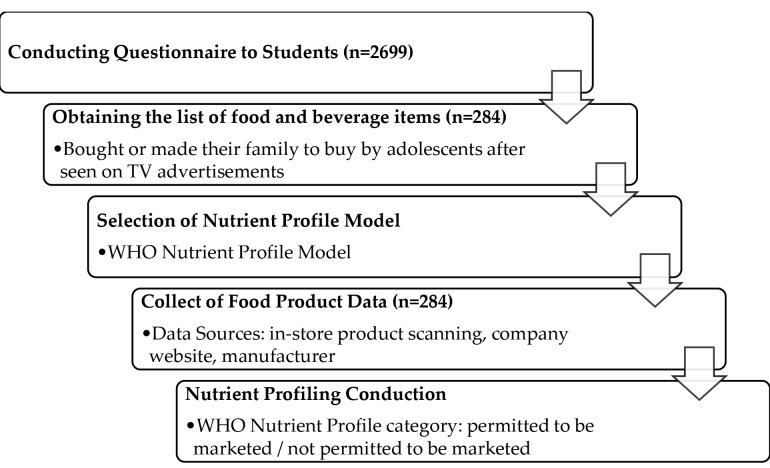
Study scheme.

 This cross-sectional survey of adolescents was carried out on 2699 students (1380 boys and 1319 girls) aged 11-16 years in eight different school settings of Altındağ district, Ankara, Turkey, in 2015. The schools were selected randomly by the Ministry of National Education, with an eye to the inclusion of participants with different socioeconomic statuses in the district, and the classes were randomly selected by administrative staff in each school. Adolescents aged 11-18 years attending the 4th to 8th grades were included in this study. However, students under 11 and over 18 years old and adolescents who did not provide written consent from their parents were excluded from the study. A scheme of the study is presented in Figure 1.

###  Adolescent dietary behaviors survey

 A questionnaire with items on demographic characteristics (gender and age), socioeconomic status (parent education and working status), television viewing habits, and purchasing tendency for foods and beverages under the influence of TV advertisements was administered to the adolescents. The three items regarding the effect of TV advertisements on one’s tendency for purchasing food and beverage in this questionnaire included “Do you intend to buy food and beverages that you see on TV advertisements?” (No, Yes); “Do you buy foods and beverages that you see on TV advertisements or make your family buy them for you?” (No, Yes); “Please write down the brand names of food and beverage products that you buy or make your family buy after you see them on TV advertisements”. Multiple responses were accepted, and children were allowed to mention up to three products. Exposure to adolescent-targeted TV advertisements was based on self-reports of TV viewing time. The dietitians took anthropometric measurements of adolescents, including body weight and height, according to standard techniques. Adolescents’ body weight was measured using TANITA Body Composition Analyser (TBF 300) to the nearest 0.5 kg, and height was measured to the nearest 0.1 cm using a stadiometer (Seca 217). Researchers referred to the Centers for Disease Control (CDC) classification for obesity definition. According to this classification, < 0.85 percentile was considered as underweight/normal weight, whereas 0.85-0.95 percentile and > 0.95 percentile were considered to be overweight and obese, respectively.^26^ Written informed consent was obtained from volunteer students and their parents before completing the study questionnaire. The study protocol was approved by the Ethics Committee of Hacettepe University, Ankara, Turkey.

###  Nutrient profiling

 The WHO nutrient profile model^18^ was launched in 2015 and developed to restrict the marketing of unhealthy foods and beverages to children and was selected to determine food and beverages’ eligibility as permitted or not permitted for marketing. This categorical model was developed based on such factors as energy, total fat, saturated fat, total sugars, added sugars, and salt content per 100 g or 100 mL. Marketing is prohibited in case these nutrients in a product exceed the determined threshold. All reported food and beverages (n = 284) bought by adolescents or their families under the influence of TV advertisements were classified into 17 categories defined in the WHO nutrient profile model.^23^ The food categories in the model included chocolate and sugar confectionery, cakes and sweet biscuits, savory snacks, beverages, edible ices, breakfast cereals, yoghurts, sour milk and cream, cheese, ready-made and convenience foods, butter, other fats and oils, bread and bread products, fresh or dried pasta, fresh and frozen meat, processed meat, fresh and frozen fruit and vegetables, processed fruit and vegetables, sauces, dips, and dressings.

###  Collection of food product data

 The information on food labels of 284 packed food and beverages was collected from three local hypermarkets by capturing the photos of the products after taking permission from the owners or managers of hypermarkets. When the food label did not include a nutrition fact label, the missing nutritional information was provided from the company websites or the manufacturer. The data extraction process recorded the product’s energy (kilocalories), protein, carbohydrate, total sugar, total fat, saturated fat, dietary fiber, and sodium (all in gram) per 100 g or 100 mL of the product.

###  Statistical Analysis

 Data were analyzed using the SPSS software (Version 22.0; Chicago, IL, USA). Descriptive statistics were computed for participants’ general characteristics and WHO nutrient profile categories, according to the nutritional content of the advertised foods and beverages. Variables were presented as mean ± standard deviation (SD) and absolute numbers (percentage) for continuous and categorical variables, respectively. Moreover, chi-square statistics were used to assess the statistically significant differences. A *P* value less than 0.05 (*P* < 0.05) was considered statistically significant.

## Results

###  Sample characteristics

Table 1 presents general characteristics of the study sample, according to purchasing status affected by TV advertisements. A total of 2699 students with a mean age of 12.9 years old completed the survey and the majority (51.1%) of whom were male. Moreover, the majority (69.8%) of students were underweight/normal weight, whereas 13.3% and 16.9% were overweight and obese, respectively. The participants’ mothers were mainly housewives (81.1%), and most fathers (92.1%) were employed. Girls were more affected by TV advertisements and prone to buy foods than boys (*P* < 0.05). Furthermore, participants in the age range of 11-13 years were more affected by TV advertisements, than those aged 14-16 years (*P* < 0.05).

**Table 1:** General characteristics of participants according to purchasing intent affected by food and beverage advertisements on TV

**Variables**	**Total (n=2699)**		**Purchasing Intent**				
			**No (n=908)**		**Yes (n=1791)**		***P *** **value**
	**Number**	**Percent**	**Number**	**Percent**	**Number**	**Percent**	
Gender							0.002
Boy	1380	51.1	503	55.4	877	48.9	
Girl	1319	48.9	405	44.6	914	51.1	
Age (y)							0.005
11-13	1808	67.0	641	70.6	1167	65.1	
14-16	891	33.0	267	29.4	624	34.9	
Mother’s education status							0.009
None	247	9.2	83	9.2	164	9.1	
Elementary school	1007	37.3	378	41.6	629	35.1	
Secondary school	587	21.7	194	21.4	393	21.9	
High school	669	24.8	197	21.7	472	26.3	
University or above	189	7.0	56	6.1	133	7.6	
Mother’s working status							0.014
Housewife	2188	81.1	762	83.9	1426	79.6	
Employed	494	18.3	139	15.3	355	19.8	
Retired	17	0.6	7	0.8	10	0.6	
Father’s education status							0.001
None	130	4.8	50	5.5	80	4.5	
Elementary school	674	25.0	271	29.8	403	22.5	
Secondary school	698	25.9	228	25.1	470	26.2	
High school	802	29.7	245	27.0	557	31.1	
University or above	395	14.6	114	12.6	281	15.7	
Father’s working status							0.326
Employed	2486	92.1	838	92.2	1648	92.0	
Retired	151	5.6	45	5.0	106	5.9	
Unemployed	62	2.3	25	2.8	37	2.1	
BMI (kg/m^2^) percentile							0.291
< 0.85	1884	69.8	624	68.7	1260	70.3	
≥ 0.85- < 0.95	358	13.3	116	12.8	242	13.5	
≥ 0.95	457	16.9	168	18.5	289	16.2	

 Participants watched television for 129.7 ± 90.8 minutes on weekdays and 174.6 ± 114.3 minutes on weekends. More than half (66.4%) of the participants sometimes eat or drink while watching TV, whereas 20.0% stated that they never eat during TV watching. Fruit, chocolate, chips, and soft drinks were reported as the most commonly consumed items during TV watching.

###  Nutrient profiling

 A total of 1877 (69.6%) participants declared that they were influenced by food and beverage advertisements on TV, and 1791 (66.4%) children stated that they bought or made their families buy those foods after watching TV advertisements. However, only 1711 (63.4%) children remembered and specified products and brands’ names. While children were asked to mention up to three products, a total of 3643 preferences and 284 different food and beverage items were recorded in this study. The most commonly reported items included cakes, sweet biscuits, and pastries (Category 2, 63.8%), chocolate and sugar confectionery, energy bars, sweet toppings, and desserts (Category 1, 44.9%), and savory snacks (Category 3, 39.6%). The least commonly reported items included sauces, dips and dressings (Category 17, 0.1%). Category 2 (26.4% of the total products), Category 1 (24.2% of the total products), and Category 3 (9.5% of the total products) were the most purchased category (Table 2). Out of the 17 WHO nutrient profile model categories, nine categories did not include any permitted food and beverages. Out of the total 284 items, only 8.5% was permitted to be advertised, according to the WHO nutrient profile model. The permitted items included dairy products (milk, kefir, ayran, and cheese), meat products (beefsteak and chicken), grains (pasta), fruits and vegetables, soup and some traditional Turkish foods (e.g., cig kofte and Turkish pizza). According to the WHO nutrient profile model, only 13.6% of the participants had bought permitted food and beverages, under the influence of TV advertisements.

**Table 2:** Purchase of advertised products distribution by categories

**Category**	**No. (%) of products**	**No. of permitted products** **WHO-NPM**	**No. of preferences ** ^a^	**% Of participants ** ^b^
Category 1	69 (24.2)	0	769	44.9
Category 2	75 (26.4)	0	1091	63.8
Category 3	27 (9.5)	1	677	39.6
Category 4-a	17 (6.0)	0	122	7.1
Category 4-b	10 (3.5)	4	63	3.7
Category 4-c	3 (1.1)	0	36	2.1
Category 4-d	23 (8.1)	0	435	25.4
Category 5	10 (3.5)	0	68	4.0
Category 6	5 (1.8)	1	38	2.3
Category 7	3 (1.1)	2	39	2.3
Category 8	5 (1.8)	2	14	0.8
Category 9	14 (4.9)	3	241	14.1
Category 10	2 (0.7)	0	1	0.1
Category 11	2 (0.7)	1	2	0.1
Category 12	2 (0.7)	2	3	0.2
Category 13	4 (1.4)	4	11	0.6
Category 14	6 (2.1)	1	20	1.2
Category 15	3 (1.1)	3	10	0.6
Category 16	3 (1.1)	0	2	0.1
Category 17	1 (0.3)	0	1	0.1
Total	284 (100)	24	3643	-

**Category 1: **Chocolate and sugar confectionery, energy bars, and sweet toppings and desserts; **Category 2: **Cakes, sweet biscuits and pastries; other sweet bakery wares, and dry mixes for making such;** Category 3: **Savoury snacks;** Category 4-a: **Juices;** Category 4-b: **Milk drinks;** Category 4-c: **Energy drink;** Category 4-d: **Other beverages;** Category 5: **Edible ices;** Category 6: **Breakfast cereals;** Category 7: **Yoghurts, sour milk, cream, and other similar foods;** Category 8: **Cheese;** Category 9: **Ready-made and convenience foods and composite dishes;** Category 10: **Butter and other fats and oils;** Category 11: **Bread, bread products, and crisp bread; **Category 12: **Fresh or dried pasta, rice, and grains;** Category 13: **Fresh and frozen meat, poultry, fish and similar;** Category 14: **Processed meat, poultry, fish and similar meats;** Category 15: **Fresh and frozen fruit, vegetables, and legumes;** Category 16: **Processed fruit, vegetables, and legumes;** Category 17; **Sauces, dips, and dressings. ^a^ Multiple choices were accepted, and participants were allowed to specify up to three products. ^b^N = 1711. Due to the multiple responses, may not sum to 100.

###  Nutritional content of advertised food and beverage products

 Food and beverages that were listed in Category 12 (fresh or dried pasta, rice, and grains), Category 13 (fresh and frozen meat, poultry, fish, and similar), and Category 15 (fresh and frozen fruit, vegetables, and legumes) were permitted, according to the WHO nutrient profile model.

 The mean ± SD energy content for products in Categories 12, 13, and 15 was determined at 354.00 ± 4.24 kcal, 193.00 ± 38.00 kcal, and 97.00 ± 34.64 kcal, respectively. The nutritional content of not permitted food and beverages is presented in Table 3. The energy, protein, fat, and carbohydrate values of foods per 100 g of the product in the most preferred category (including cakes, sweet biscuits, and pastries) were determined at 441.96 ± 97.87 kcal, 5.98 ± 2.59 g, 20.81 ± 7.89 g, and 58.42 ± 17.86 g, respectively. Energy, protein, fat, and carbohydrate values for chocolate and sugar confectionery, energy bars, and sweet toppings and desserts category were determined at 436.12 ± 132.82 kcal, 6.19 ± 3.53 g, 21.63 ± 12.59 g, and 54.10 ± 19.65 g, respectively. Moreover, these two categories are not permitted in the WHO nutrient model, regardless of their nutrient content. Due to the fact that the permitted salt content is 0.1 g per 100 g of the product, according to the WHO nutrient profile model, 26 food products in Category 3 (savory snacks) were not permitted to be advertised. The mean ± SD sodium content of items in Category 3 was found to be 1.63 ± 0.55 g. It should be noted that Category 4a (juices) and 4c (energy drinks) are not permitted, based on the WHO nutrient model, regardless of their nutrient content. However, the items in Category 4b (milk drinks) could be allowed to be advertised in case the total fat of the product does not exceed 2.5 g/100 g. The mean ± SD total fat amount for not permitted products in Category 4b was estimated at 3.05 ± 0.12g. In the cheese category (Category 8), the total fat in 100 grams of the product must not exceed 20 grams, based on the WHO nutrient profile model. However, in this study, the mean ± SD amount of fat in not permitted cheeses was 29.33 ± 7.33 g/100g. Regarding Category 9 (ready-made and convenience foods and composite dishes), energy, total fat, and saturated fat values must not exceed 225 kcal/100 g, 10 g/100 g, and 4 g/100 g, respectively, according to the WHO nutrient profile model. Nonetheless, the mean ± SD values for energy, total fat, and saturated fat were determined at 414.73 ± 408.61 kcal/100 g, 24.52 ± 28.72 g/100 g, and 5.40 ± 0.57 g/100 g, respectively, for not permitted products in Category 9. In addition, saturated fat must not exceed 20 g/100 g in Category 10 products (butter and other fats and oils). However, the mean ± SD amount of saturated fat for Category 10 products was 50.65 ± 0.92 g/100 g, and the mean ± SD amount of total fat for Category 14 and Category 16 products was estimated at 23.80 ± 12.87 g/100 g and 10.21 ± 11.79 g/100 g, respectively.

**Table 3:** Nutritional values of not permitted foods

**Category**	**n**	**Energy (kcal)**		**Protein (g)**		**Fat (g)**		**Saturated Fat (g)**		**Trans Fat (g)**		**CHO (g)**		**Sugar (g)**		**Sodium (g)**		**Fibre (g)**	
		**Mean**	**SD**	**Mean**	**SD**	**Mean**	**SD**	**Mean**	**SD**	**Mean**	**SD**	**Mean**	**SD**	**Mean**	**SD**	**Mean**	**SD**	**Mean**	**SD**
Category 1	69	436.12	132.82	6.19	3.53	21.63	12.59	12.45	7.01	0.01	0.01	54.10	19.65	3.07	2.30	0.33	0.35	3.07	2.30
Category 2	75	441.96	97.87	5.98	2.59	20.81	7.89	10.79	5.19	0.04	0.05	58.42	17.86	2.90	1.41	0.61	0.53	2.90	1.41
Category 3	26	480.42	58.41	10.34	6.95	23.40	10.14	6.57	4.28	0.07	0.07	57.40	14.84	4.74	3.03	1.63	0.55	4.74	3.03
Category 4-a	17	105.30	130.94	1.14	2.56	3.37	9.31	1.00	3.61	-		13.63	10.38	0.56	1.12	0.02	0.07	0.56	1.11
Category 4-b	6	108.82	47.84	3.88	1.33	3.05	0.12	-	-	-		10.23	8.80	-	-	-	-	-	-
Category 4-c	3	38.06	19.93	-	-	-	-	-	-	-		9.33	4.76	-	-	0.11	0.10	-	-
Category 4-d	23	69.68	96.59	0.92	3.04	0.79	2.44	0.16	0.49	-		14.40	18.37	0.57	2.05	0.13	0.35	0.57	2.05
Category 5	10	227.70	53.50	3.10	0.57	11.44	4.46	8.13	4.35.	-		24.22	6.95	1.05	0.35	0.13	0.12	1.05	0.35
Category 6	4	361.33	34.91	7.15	2.42	6.72	8.25	4.25	6.53	-		64.93	24.05	6.53	1.33	0.62	0.70	6.53	1.33
Category 7	1	43.20	-	2.00	-	1.80	-	1.26	-	-		3.5	-	-	-	0.26	-	-	-
Category 8	3	458.09	145.25	22.16	5.76	29.33	7.33	-	-	-		26.30	22.65	-	-	3.21	0.90	-	-
Category 9	11	414.73	408.61	16.33	13.35	24.52	28.72	5.40	0.57	-		49.73	51.78	1.46		3.46	3.03	1.46	-
Category 10	2	742.00	-	0.30	-	82.00	-	50.65	0.92	-		0.60		-		-	**-**	**-**	**-**
Category 11	1	520.00	-	6.50	-	32.30	-	-	-	-		58.60	-	9.90	-	0.04	-	9.90	-
Category 14	5	278.60	122.59	13.60	1.516	23.80	12.87	-	-	-		2.90	1.14	-	-	2.72	0.38	-	-
Category 16	3	368.00	71.08	10.43	10.30	10.21	11.79	-	-	4.30		59.70	13.12	4.43	3.15	0.18	-	4.43	3.15
Category 17	1	136.00	-	1.20	-	-	-	-	-	-		24.00	-	-	-	-	-	-	-

*Category 12, Category 13, and Category 15 were permitted categories according to the WHO nutrient profile model.

## Discussion

 This study assessed the nutritional content of food and beverages advertised on TV based on the WHO nutrient profile model. The study results revealed that girls were more affected by TV advertisements and tended to buy the advertised foods more than boys. Harris et al^27^ tested the relationship between exposure to food advertisements and snacking on available food in a randomized control trial and reported that males eat significantly more after exposure to food advertisements, compared to females. Contrary to the study conducted by Harris et al,^27^ Anschutz et al^28^ reported that females showed a significantly higher tendency for unhealthy snack food intake, following the exposure to food advertisements, compared to males. In addition, a systematic review of Mills et al^29^ regarding the effects of food advertising on food-related behavior, attitudes, and beliefs demonstrated that the effect of gender on getting influenced by food advertisements is inconclusive. It was also found that younger adolescents were more prone to buy food products, following exposure to advertisements. Marsh et al^30^ showed a link between screen time and increased food and soda intake in children rather than adolescents and young adults. Consequently, specific populations may be at increased risk of excessive energy intake when engaged in screen-based behaviors.

 It is widely accepted that long-term TV viewing and leisure-time sedentary behaviors during adolescence lead to immediate and long-term health outcomes, including increased risk of obesity.^18^ Dalton et al^31^ stated that children spent 1-3 h daily watching TV. Fletcher et al^32^ demonstrated that adolescents with a mean age of 12.9 years watched TV for 3.2 h/day. In addition, Barker et al^33^ showed that TV viewing time for boys and girls was 121.8 ± 69.9 min/day and 111.0 ± 66.1 min/day, respectively. In line with the results of these studies, the findings of the present study showed that TV viewing time of adolescents was 129.7 ± 90.8 minutes and 174.6 ± 114.3 minutes on weekdays and weekends, respectively.

 In this study, out of the total 284 food products, only 8.5% of them were permitted by the WHO nutrient profile model. In the same line, Patino et al^11^ reported that more than 60% of the foods advertised in Mexico did not meet any nutritional quality standards. Korosec and Pravst^24^ reported that 96% of food advertisements in Slovenia did not pass the criteria for obtaining advertisement permission, according to the WHO nutrient profile model. This result was also supported by a recent report from Turkey, showing that 78.8% of the food products were not suitable for marketing to children.^34^

 The obtained results in this study supported the conclusion that TV viewing has been associated with unhealthy food choices, depending on the exposure to food advertisement, as indicated in the previous studies.^31,35-38^ Based on the evidence, acute exposure to food advertisement increases food intake in children, though not in adults. Consistent with the results of previous studies involving content analysis of food advertisements,^39-41^ the results of the present study revealed that most of the foods analyzed by the WHO nutrient profile model were high in energy and low in nutrient density products. The most significant proportion (26.4%) of these advertisements was related to cakes, sweet biscuits, and pastries. The second most prevalent (24.2%) advertisements were related to chocolate and sugar confectionery, energy bars, and sweet toppings, and desserts.

 Based on the results of another study, chocolate (20.7%) and edible ices (18.8%) were the most advertised foods on TV in Turkey, which confirmed the results of the present study.^34^ However, in contrast to the result of the present study, participants were less affected by the advertisements of high nutrition value foods (e.g., fresh and frozen meat, poultry, fish, fresh and frozen fruit, vegetables, and legumes.

 WHO reported that foods high in fat, sugar, and salt were marketed to children.^23^ The absence of vegetable and fruit advertisements was also reported in other countries, such as New Zealand and Argentina.^39,41^ In Mexico, it was found that the most nutritious categories (i.e., fresh and frozen fruits, vegetables and legumes, meat, poultry, and fish) represented less than 1% of advertised products.^11^

 Savory snacks (9.5%) were another group of products preferred by adolescents under the influence of TV advertisements. These results were consistent with those of other studies on the marketing of sweet drinks, salted snacks, and fast food to children.^41-43^ Looking at advertisements (e.g., juices, milk drinks, energy drinks, and other beverages) in general showed that only 4 out of the 43 beverages were in the permitted group, according to the WHO nutrient profile model. These four beverages were in the milk drink category. Given that carbonated beverages not only have adverse effects on oral health and are linked with increased risk of obesity, they are also associated with reduced milk consumption, which have a significant adverse effect on calcium levels. Therefore, this is a particular concern from the public health context.^44-45^

 Robust data support the fact that the foods advertised to children were high in energy content. A study conducted in Mexico found that the foods advertised during cartoon shows had the highest energy content (367 kcal).^11^ Another study showed that foods advertised on TV were high in calories, fat, and carbohydrates.^46^ In line with the previous studies, the results of this study showed that the content of energy, fat, and carbohydrates in the advertised foods is high. According to WHO, one of the marketing recommendations is to reduce the impact of advertised foods high in saturated fatty acids, trans-fatty acids, free sugars, or salt/sodium on children.^23^

 Regarding the limitations of the present study, one can refer to the fact that the cross-sectional nature of this study may have affected the representativeness of the data, due to the sampling method adopted in this study. Moreover, it should be noted that nutrition labeling was not mandatory in Turkey during this study; therefore, available data were mostly insufficient for the application of the WHO nutrient profile model. One of the strengths of this study is that the data collected here can provide valuable information about TV food marketing in Turkey. This study is an important initial exploration of the relationship between the nutritional quality of advertised food and beverages and adolescents’ food choices.

## Conclusions

 Based on the obtained results, the great majority of foods purchased following the exposure to advertisement were linked to energy-dense foods and did not comply with any nutrition quality standard. This study confirmed the need for regulatory marketing restrictions to prevent the marketing of unhealthy products, including changing food advertisements times (away from peak viewing times) and using the WHO nutrient profile model to restrict unhealthy food advertising on TV. Future studies are warranted to further focus on the exposure to unhealthy food advertisements and the power of advertisements through other communication tools, including product placement within programs, social media, and the internet.

## Acknowledgments

 The authors would like to thank the study respondents for their participation in this study.

## Authors’ Contribution

 ZBD, RN, AA, and AA contributed to the study’s design; EB and KI contributed to carrying the study out; ZBD, DD and EI contributed to analyzing the data; ZBD, DD and EI writing the article. All authors read and approved the final manuscript.

## Conflict of Interests

 The authors have no conflict of interest regarding the publication of this study.

## Funding

 none.

**Highlights**

- Adolescents were affected by TV advertisements, and girls were more affected by TV advertisements than boys (*P* < 0.05).
- The tendency to buy food products increased in younger adolescents under the influence of advertisements.
- Among all the food product items advertised (n = 284), only 8.5% was permitted by the WHO nutrient profile model.
- The majority of the foods analyzed by the WHO nutrient profile model were high in energy and low in nutrient density.
